# Understanding adverse incident responses in mental health care: a qualitative study of systems-based patient safety practices

**DOI:** 10.1136/bmjopen-2025-104863

**Published:** 2025-11-09

**Authors:** Alexander Challinor, Kathryn Berzins, Oladayo Bifarin, Nina Anderson, Panchu Xavier, Pooja Saini, Esmaeil Khedmati Morasae, Rajan Nathan

**Affiliations:** 1 Mersey Care NHS Foundation Trust, Liverpool, UK; 2 University of Liverpool, Liverpool, UK; 3 University of Central Lancashire, Preston, UK; 4 Liverpool John Moores University, Liverpool, UK; 5 National Institute for Health and Care Research, London, UK; 6 Cheshire and Wirral Partnership NHS Foundation Trust, Chester, UK; 7 Health Services Research, Liverpool John Moores University, Liverpool, UK; 8 University of Exeter, Exeter, UK

**Keywords:** safety, health & safety, mental health, psychiatry

## Abstract

**Abstract:**

**Background:**

A key part of the patient safety system is how it responds to and learns from safety incidents. To date, there is limited research on understanding system-based approaches to investigating incidents that occur within this complex interacting system.

**Objectives:**

The aims of this study were to qualitatively explore mental health professionals’ perceptions of patient safety incident investigations; to understand the impact of the transition to systems-based approaches and to explore the influence of different elements of the system on the goals of patient safety.

**Design, setting and participants:**

The qualitative study involved 19 semi-structured interviews with professionals working within the patient safety system across two mental health National Health Service trusts. The data were analysed using thematic analysis.

**Results:**

Those interviewed identified that a change in approach to incident investigation, from root cause analysis to systems-based, would lead to rigorous investigations that are effectively linked to learning. Over time, this was described as a contributory factor to reducing feelings of blame and positively influencing safety culture. There were considerations of potential negative effects from a systems-based approach, such as the shifting rather than elimination of blame, and the possibility of missing individual poor practice. The findings identify the presence of several interdependencies across the system that could have a positive or negative influence on the outcomes of incident responses.

**Conclusions:**

This study demonstrates that the interdependencies within the system and our limited understanding of safety in mental healthcare introduces complexity and uncertainty to incident investigation outcomes. This is likely to impact on safety incident responses and learning, where acknowledging and evaluating this complexity is likely to reduce any potential negative outcomes that exist.

**STRENGTHS AND LIMITATIONS OF THIS STUDY**

- This study adopted a purposive sampling approach to ensure a range of professionals were interviewed across the safety system.
- The research team comprised professionals from various fields, including clinician-researchers, resident doctors, psychologists, nurses and senior patient safety managers.
- The project used an advisory group of public and patient involvement and engagement representatives who helped shape the aims, recruitment, interview schedule and the findings for the project.
- Although the exploration and mapping of the safety systems complexity was an important finding, this complexity means that certain stakeholders in the system may have been missed from the interviews conducted.

## Introduction

 Patient safety is a healthcare discipline aimed at creating a trustworthy system for care delivery that minimises the incidence and impact of preventable harm.^1^,^3^ Occurrences of potential and/or actual harm in healthcare are known as patient safety incidents. The primary aim of investigating these incidents is to gain insights into how they occurred with a view to reducing the likelihood of future recurrence. Incident investigations seek to understand how various contributory factors intersect and may lead to either unsafe or safe care.^4^ Individual patient safety processes (such as reporting, investigating, learning and improving patient outcomes) activated following serious adverse incidents (eg, patient suicide) are interconnected and not standalone steps. As such, these processes are embedded within a complex system of interdependent actions, enacted by individuals who are influenced by an array of implicit and explicit influences.^5^

Despite the growth of interest in, and understanding of, safety research across healthcare, there has been minimal exploration into patient safety in mental health services. There is limited research on patient safety in mental healthcare, and even less on system-based approaches to investigating incidents in mental healthcare.^6^,^8^ Those receiving and delivering care in mental health services do face some similar types of harm to those in other areas of healthcare, meaning that this empirical work is transferable to a degree. However, the behaviours associated with psychiatric presentations (eg, self-harm and/or violence to others) and the interventions aimed to manage these (eg, risk assessment tools, coercive measures) add further layers of complexity to patient safety.^9 10^ Additionally, the notion of risk is thought to differ in mental health services in comparison with physical healthcare, for example, it is not just that we are concerned with the adverse effects of the pathology and treatment, but also with the possibility of an as yet unrealised state of consciousness arising at some point in the future.^8^

Within healthcare systems, patient safety should be understood as a multifaceted intervention designed to collectively reduce preventable harm.^11 12^ Specifically, the National Health Service (NHS), UK, has begun to recognise the need for this. In March 2020, NHS England, UK, launched a new patient safety strategy, the Patient Safety Incident Response Framework (PSIRF).^3 13^ This strategy aimed to promote safety culture through patient safety systems, highlighting the need for ‘insight’, an approach to improve understanding of safety by drawing from multiple sources of patient safety information.^3 13^

This is a critical strategic shift, where PSIRF provides greater flexibility in investigating and learning from incidents. The changes are summarised as (i) a shift away from investigating all incidents above a certain threshold to a more flexible proactive approach to learning, (ii) a change in the approach to investigation (system-based, defined with quality, and based on opportunity for learning) and (iii) changes to the experiences of those affected by incidents (patient, family, carers, staff).^14^ Evidence suggests that the implementation of PSIRF will be challenging, owing to the multilevel complex healthcare ‘system’, where little is known about how stakeholder groups and regulators both shape, and are shaped by, safety incident investigation and responses.^14^

Patient safety is thought to be a good entry point to use system-based practice for incident investigation.^15 16^ The new approach, PSIRF, introduces a broader systems-based approach to incident investigation and responses, a shift away from the traditional linear cause effect model of root cause analysis (RCA), which focuses on individual human factors and errors. This shift aims to replace ‘blame culture’ or defensive practices with a ‘learning culture’, encouraging a more constructive view of patient safety that addresses systemic factors rather than isolating individual fault.^13^

The systems approach to patient safety incident investigation should aim to answer fundamental questions about the wider system in which people operate, the opportunities for risk and to develop strategies designed to improve quality of care and mitigate harm.^10^ However, this may be difficult if we lack an empirical understanding of the ‘system’ of patient safety and its complexity, especially within the context of mental healthcare delivery. Complexity and systems thinking can develop an understanding of what the patient safety system does, how it succeeds but sometimes fails.^8^ Developing a qualitative understanding of the system components, incident investigation processes and the inter-relationships and interactions of the safety system will yield important findings for safety strategy and future research.

An exploration of how the newly introduced systems-based incident investigation process has influenced how organisations investigate, respond and learn from incidents across the complex system is warranted. Although some specific elements of the response to adverse incidents have been examined, no previous empirical research has set out to study the complex interacting multi-level system within which these elements are situated.^11^ Given the inherent complexity and the epistemic uncertainty of the patient safety system in mental healthcare, it is imperative to evaluate and explore the operationalisation and effectiveness of system-based incident investigations within the complex system.^8 10^

## Aims

The aim of the study was to qualitatively examine the perceptions of participants on safety incident responses and investigations in mental healthcare, specifically, to better understand the impact of transitioning to safety-based incident investigation and how different elements of the system influence the goals of patient safety.

## Methods

### Study setting and design

This study was conducted across two NHS trusts in England, UK. A mental health NHS trust is an organisation that provides health and social care services for people with mental health disorders within a defined geographical area.

The study was completed with a qualitative design applying individual semi-structured interviews and was guided by the Standards for Reporting Qualitative Research checklist (online supplemental material A).^17^ The research itself adopted a social constructionist and interpretivist perspective, with the aim to understand a particular concept through the meaning that individuals ascribe to the concept based on their personal experience in a specific setting.^18^

### Recruitment

Participants were recruited using a number of methods. Within the two trusts, two data collectors met with the Director of Patient Safety and the Associate Director of Patient Safety in each respective NHS trusts. This discussion provided a recruitment map that identified departments/teams involved in safety incident investigations within each organisation. Convenience sampling was initially used where the study team contacted the identified senior leader who disseminated the study information packs and research contact details within their department/team. Departments/Teams included the patient safety team, the incident and risk team, nursing and quality, social health and communities (eg, social work) and medical services (eg, frontline clinicians, clinician-managers). People who were interested in participating contacted the researcher who then provided them with a Participant Information Sheet.

Purposive sampling was also used across each NHS trust to ensure the study obtained a range of professionals working across each organisation, and across different teams within the organisations. Staff were chosen from the identified team lists and directly contacted by the research team for involvement in the study. If the invited individual declined or did not respond, another person was chosen with a similar role.

The researcher offered various methods of contact at the participants’ convenience to discuss the study further. For participants who agreed to take part, an interview was scheduled in a format (virtual or face-to-face), location and at a time convenient to them, with informed consent obtained prior to participation.

The research team met with directors of patient safety to review the recruitment progress. A decision to cease sampling was made when the sample size was thought to be sufficient to capture a range of experiences across the safety system and to address the research aims. Two researchers were present across all stages of sampling, collection and analysis, embedding an iterative process to assess data saturation. The thematic analysis described below continued until themes identified were consistently repeated and no new information of relationships between themes emerged.^19 20^

### Data collection

An interview schedule was designed by the research team, with support from a team member who holds expertise as a Director of Patient Safety within an NHS trust. The interview schedule was also developed with patient and public involvement. The interview format covered areas of the participants’ role and understanding of the safety system; their experiences of the processes within the system; their interplay with the incident investigation and response processes; learning and improvement from patient safety incidents; culture and patient safety and inequalities within the system. The interview schedule can be found in online supplemental material B.

Semi-structured interviews were conducted face-to-face at the participants’ workplace or on a virtual platform. Three researchers conducted the interviews. The positioning and reflexivity of the researchers were considered throughout the research process, and this is detailed further in online supplemental material C.

No time limit was placed on the interviews to allow for open exploration and natural conclusions to be drawn. Interviews lasted between 30 and 60 minutes and took place between June 2023 and September 2024. Data were audio recorded, transcribed to ensure participant anonymity and stored on secure NHS servers.

### Data analysis

Qualitative data were analysed using the Framework Method involving seven stages.^21^ This was selected as the most appropriate method due to the large data set that required comparisons with and between interviews. Two researchers who were involved in data collection independently analysed the data. The authors familiarised themselves with the data set and coded transcripts independently. An initial list of codes was developed, guided by the initial review and the theoretical topics within the interview guides. This identified themes and subthemes within and across codes, shaping an analytical framework. Categories within the framework aligned with the research aims (systems-based investigations and impact of system elements on safety incident investigation). The two researchers compared the themes, developing the coding framework and matrix. The interpretations of the data were shared and reviewed between the researchers to agree and finalise broader themes.

Additional expert-led member-checking discussions were held with the Director of Patient Safety within the research team to provide expert oversight and to triangulate the findings with other researchers during the analysis and interpretation process. The final themes were then shared with the research team for further consideration and refinement. Quotations were selected for their representativeness of the identified themes and were reviewed by the research team to reach consensus on their inclusion.

### Patient and public involvement

The project used an advisory group of public and patient involvement and engagement (PPIE) representatives who helped shape the aims, recruitment and interview schedule for the project. Five PPIE representatives were recruited, all of whom had experience as a patient/carer in mental healthcare. The feedback from the PPIE representatives is also embedded within the data analysis. The PPIE group will be used to help guide future outputs and research from this study.

The original protocol for the study can be found in online supplemental file D.

## Results

A total of 19 professionals participated in the semi-structured interviews across two NHS trusts. Ten participants were recruited from one NHS trust (labelled IV1–IV10 within quotations) and nine participants from the second NHS trust (labelled IV11–IV19). The sample included professionals working within the patient safety system and across the different levels of healthcare organisation. Table 1 shows the range of professional roles of the participants involved in the study. The roles were grouped into broader categories across the system to maintain confidentiality. All participants reported that their roles involved experience of working within the patient safety system in a mental healthcare organisation. Six of the participants held frontline clinician roles in addition to those listed in table 1.

**Table 1:** Professional roles of the participants involved in the study

System group	N*=***19**
Patient safety team	5
Governance, risk and investigations managers	4
Clinical service leaders	5
Operations managers	5

### Findings

Box 1 portrays the five themes that were identified within the data, along with a brief description of the findings.

Box 1 **Themes identified from the analysis with a brief description of the interview findings**

**Theme 1: the dichotomy of incident investigation methods**

*Participants described aspects of different incident investigation approaches as mutually exclusive, describing that root cause analysis often overlooked systems-based factors, while systems-thinking approaches tended to neglect human error*.

**Theme 2: the influence of system interdependencies**

*Participants described how different elements of the safety system can interact to influence the goals of patient safety incident investigations*.

**Theme 3: safety culture and feelings of blame**

*This theme identifies the perceived changes to safety culture resulting from a change in incident investigation approaches*.

**Theme 4: redistribution of incident accountability**

*Those interviewed noted a potential shift in accountability from the frontline to middle managers following the change to systems-based approaches*.

**Theme 5: operationalisation of learning from incident investigation**

*Within this theme, participants identified the challenges in implementing learning and facilitating effective change to prevent further harm*.

### The dichotomy of incident investigation methods

Participants described aspects of systems thinking and RCA as mutually exclusive, noting that the RCA approach ignores systems-level recommendations, while systems thinking may not adequately account for potential human error. This was more commonly found in the participants who were not actively involved in the ‘industry’ (IV6) of reviewing and investigating incidents, that is, frontline clinicians and managers. All participants interviewed expressed that system-based investigations and responses were more robust, reliable and comprehensive. There were no participants that identified RCA as a preferred approach to incident investigation.

*So, a prime example for me is that under the root cause analysis framework and methodology that we previously used, investigations weren’t as robust. I feel that since we've moved to the systems-based approach, they are far more robust and in-depth.* (IV3)

Participants noted the better-quality outcomes and recommendations from systems-based investigations, linking these to safety culture and learning from incidents (themes 3 and 5). The quality was attributed to the benefits for the entire organisation, rather than the isolated team in which the incident occurred, as well as developing co-produced learning that is likely to have wide applicability to the clinical service.

*I can’t see how any single investigation of an index incident that then goes on to make recommendations at trust level. Is it justifiable really because you know, it [RCA] hasn’t considered the way we deliver practise wider than the team in which the incident happened, whereas the new PSIRF, if in theory, does allow for that.* (IV5)

*We’re really trying to unpick the systems behind. So, there’s, there’s very much more emphasis on the action plans being a coproduced with the people who are going to be delivering the actions, which is excellent step forward.* (IV2)

Participants described how systems thinking may miss human error if that was the causal explanatory factor for the incident, an outcome that RCA investigations would not miss. There were concerns that by investigating systems-based factors, the incident investigation may miss the decision-maker (the human) as the cause of an event rather than a contributory factor existing within a system. The disadvantage of ‘*missing the bad apple*’ (IV5) was linked to patient safety culture and effective learning within the organisation.

*One of my personal frustrations when I… experienced working within a just culture… in that way of working…, I felt it had gone too ‘one way’. So, there may be occasions where actually people have done wrong.* (IV7)

*Is there any suggestion of, you know, poor practice malpractice then that would be the point of picking that up. Do we do that robustly enough? I’m not so sure.* (IV5)

*To make sure the true learning is happening because what we are seeing is similar incidents, similar incidents still happening, you still got to bring them back to some of those basics as well. So, you don’t want to move too far away. I’m not saying we blame people, but you have to remind people of their own responsibility and accountability as well.* (IV13)

### The influence of system interdependencies

Exploring the influence of the whole system and its parts within the interviews revealed a theme in which participants identified different interdependent elements of the system and how they interact to try to achieve the same safety goals. This theme stemmed from the complexity of the patient safety system, and specifically how this complexity influenced the overall function of the system processes. This occurred within various system parts, levels and relationships.

**Figure 1:**
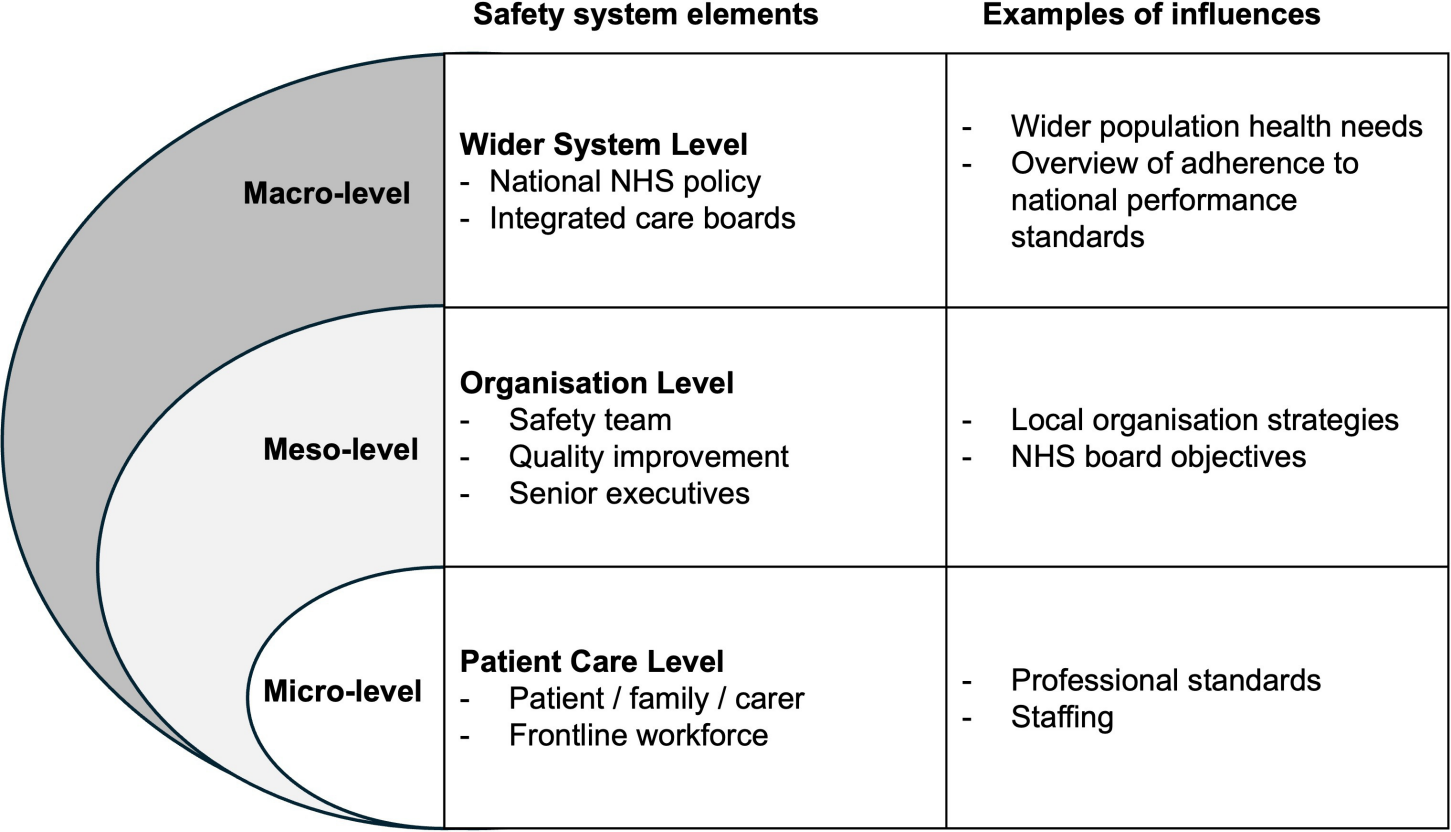
Healthcare organisation levels grouped into macro-level, meso-level and micro-level system elements, with examples of potential influences from each level. NHS, National Health Service.

The interviews revealed different expectations both across system levels and within the organisational patient safety system pathways. The analysis has placed the participants’ identified interdependencies into one of three levels of the healthcare system: the macro-level, involving system-wide approaches with a focus on strategy/policy/infrastructure; the meso-level, the organisation levels involving local systems, pathways, services and micro-level, comprising the patient care level.^22^ Interviewees shared both advantages and disadvantages of the multiple parts of the patient safety system and the impact of interdependencies across different levels of the system. Figure 1 provides a pictorial representation of the system levels.

At the macro-level, participants expressed the effect of external system-wide influences that focus on organisation strategy and policy, with downstream processes influencing pathways at the local organisational level. The input of these elements was thought to be necessary; however, it was sometimes identified to cause a negative impact. Examples of the external influences included Integrated Care Boards (*ICB*) (IV1) and the Care Quality Commission (*CQC*) (IV10). Participants described how these external influences can ‘*shape(s) the strategy*’ (IV10), alter the nature of incident investigations to meet the elements ‘*questions*’ (IV1) and place additional demand or ‘*pressure*’ (IV17) on the local system.

*I suppose, held accountable by some of these organisations [CQC, ICB], so it helps and shapes the strategy and our focus based on what the accountability and what the ask is.* (IV10)

*I think the people you know, I saw for now thinking trying to predict what those questions are, that the ICB are going to ask to answer all of those. And rather than focusing on what can we do to make this patient safe or these services safe now and action in those it’s gone a bit broader.* (IV1)

*It’s just competing priorities, and I think there’s competing understandings as well. I think a lot of it’s about time scales, isn’t it? … You know there would be that pressure of what we’ve [the local organisation] seen is this, where is it [the incident response outcome], you know for escalating externally [to the agency].* (IV17)

Most participants expressed the effect of expectations across the organisation at the meso-level (ie, across the local NHS organisation). For example, the participants identified that the expectations and outcomes of incident investigation may be different between the trust executives, the patient safety team, the incident response team and the clinical workforce on the ground. These differences in expectations were largely felt as criticisms or scrutiny as the process/pathway within the system moved downstream (ie, down the hierarchical NHS trust leadership structure).

*I do feel that we can take things to trust panel and it’s very, you know routine…we’re saying we've identified this learning and it’s just constantly, but there’s more you could have done with this.* (IV12)

The participants identified the influence of the different organisational levels on the safety incident investigation and response processes. The input from specific local organisational teams or elements can influence the processes downstream (ie, from the senior executive management to the frontline workforce). The usual processes began with an incident being recorded by the clinical team. This incident is then reviewed and investigated by the team/service within the organisation responsible for incident investigation. Depending on the seriousness of the incident, an incident response will move upstream in the hierarchy of the organisation, having input and feedback from the patient safety and trust board senior management teams. The participants observed that the patient safety system may not function as intended (ie, in keeping with PSIRF) when an incident moves upstream to senior trust executive oversight and opinion.

Participants identified that upstream in the meso-level, the senior management felt that more accountability was required for individual human factors than system-based factors. This perspective was thought to influence safety culture and operationalisation of wider system-based learning.

*I know that I would have on some occasions got push back [from trust executives] if I’d put recommendations which were kind of widespread recommendations rather than well, individual supervision with the nurse.* (IV6)

*Senior staff within the organisation around about professional accountability, calling staff to account and I’m not saying it’s a blame culture but you’re hearing conversations potentially in unguarded moments, or even in a committee where their concept of what should happen is very different.* (IV14)

*There’s more ‘you could have done this’. ‘You could have done that’…. And I do feel that the corporate teams are far removed from what is actually going on the… ground.* (IV12)

However, interviewees did note advantages to a system with multiple interdependencies across the meso-level local organisational pathway, including a broader view on the incidents and how these differing perspectives can help incident investigation processes and outcomes.

*You’ll get an incident that you think, ‘oh, I’m not sure’ of. And then you’ll have a very different view from what… the central patient safety team say it has, what the issues are… you do get that, and you know, we’ll talk through that. We have that challenge. That’s fine. And it is a healthy challenge.* (IV17)

*There’s this nuance that sometimes one committee might miss, but someone else might pick up and you… really need everybody sort of feeding in.* (IV13)

The PPIE work conducted revealed that there was trust in clinical professionals and their involvement with safety incident investigation. However, the PPIE representatives noted that they would lack trust that an appropriate outcome would be reached when more senior trust executives were involved. The participants in the study revealed that embedding a system-based culture organisation wide, alongside accessibility and authenticity from corporate stakeholders, would reduce the potential negative effects highlighted in the interviews.

### Safety culture and feelings of blame

A common theme identified by the participants was the influence of safety culture within the organisation, particularly regarding how the shift to system-based investigations might impact organisational culture. Safety culture was referred as needing change for PSIRF to be effective, while the adoption of PSIRF was also expected to drive subsequent cultural change. Most participants were expectant and/or hopeful that the change in patient safety strategy would improve culture. This change was expected to encourage reporting by frontline staff and to reduce feelings of blame among those involved in incident investigations.

*I think that because we are now working with the systems-based approach and it’s a culture change as we know, culture is very difficult to change in an organisation. But I do feel that we are making those steps now and those roads to staff not feeling perhaps intimidated by the process or worried by the process.* (IV2)

Some participants felt that, despite a system-based investigation and corresponding recommendations, individuals involved in an incident investigation are still likely to experience feelings of blame.

*Then the team will be interviewed. They might get very defensive and yes, there are connotations of blame. So, in terms of psychological safety, for example, it doesn’t nurture or encourage that at all.* (IV5)

*I think if you were to ask the consultants you know and we’ve spoken about, consultants have been affected. Those who’ve had reports that were system-based investigations. I’m not sure they’d say that it felt blameless.* (IV8)

### Redistribution of incident accountability

Some participants thought that the systems approach had a change in load distribution within the complex system, that is, the outcomes from an incident investigation shifted from the frontline (as perceived to occur in RCA) to another element in the safety system. The participants described a shift of accountability being brought upstream in the patient safety system to a middle manager (eg, nursing matrons, clinical service leaders). This was in comparison with previous patient safety strategies (Serious Incident Framework, RCA), where recommendations were often placed on individual professionals delivering direct clinical care.

Consequently, there were considerations of how system-based investigations may be impacting downstream outcomes of accountability, for example, a negative influence from team managers on the frontline workforce. There was consideration of how this shift in accountability may impact safety culture and the willingness of clinical teams to raise concerns.

*I don’t feel that blame stay with the staff on the front line now. I feel like the blame sort of comes and hits the senior management now. Which is? I mean it should be that way. Don’t get me wrong, but I feel it’s very much something happened and straight away it’s on the management’s rather than the staff on the ground.* (IV14)

*I think sort of it was down to pressure on the frontline people’s, you know, discourage from reporting certain things because or trivialising things. A lot of the lower banded staff just didn’t feel able to speak up at all for fear of kind of retribution: that they’re given the, you know, the rubbish shifts or some people say, you know, to just exclude it. People stop talking to them. Just seems a troublemaker.* (IV18)

### Operationalisation of learning from incident investigation

Most participants shared their views on how to translate the incident investigation process into effective learning and the challenges of sustaining and embedding that learning over the long term. The participants revealed that due to the volume of incidents that do occur, the governance teams and clinical teams can often become focused on the process of incident investigation, which some participants referred to as an ‘*industry*’ (IV6). This meant that resources and time were thought to be less available to share or implement any learning following the investigation.

*We get lots and lots of data, lots of information around where things go wrong. What I think we’re not so good at is translating that information, that data, then into improvement activity*. (IV18)

*It ends up in lots and lots of meetings everywhere, and actually, the amount of data that is generated is actually immense and it’s handling the capacity or creating that capacity within teams to be able to analyse the data and learn from it.* (IV10)

*I think it’s become a bit more of a tick box exercise rather than really thinking about the learning.* (IV1)

When learning did follow on from investigation, the participants thought it was challenging to ensure that the learning was effective and had a long-term impact on quality of care and patient safety. Reasons, when provided, were thought to be associated with the complexity of the system and meeting the different expectations of the interdependencies as well as the limited resources needed to drive meaningful change. The participants identified that the incident response would result in an initial learning outcome, for example, ‘*audit*’ (IV7), a change in ‘*policy*’ (IV8). However, whether that initial learning leads to effective change to prevent further safety incidents was questioned.

*One of the things that I’ve been thinking about more recently is how do we make sure that that remains [learning].…I think we’re good at reacting and putting systems and audits and all of that in in the short term, but then through time it gets lost.* (IV7)

*You’ve got the policy the way you want it based on an incident that that makes sense, but I think there’s probably a need to pay more attention to whether the policy impacts practice.* (IV8)

## Discussion

### Main findings

This study is, to our knowledge, the first to empirically examine the real-world operationalisation of the Patient Safety Incident Response Framework in mental healthcare. Participants in this study reported that the change in approach to incident investigation is likely to lead to beneficial change. The systems approach to investigation was thought to be more robust and more effectively linked to learning. These perceptions are supported by the empirical evidence base that drove the shift to PSIRF in the NHS.^3 14^ There were considerations of potential negative effects from a systems-based approach, such as the shifting rather than elimination of blame, and the possibility of missing individual poor practice. Despite improved links to learning, participants described difficulties in translating identified learning into effective change. Resource implications of the incident ‘industry’ were an explanation, possibly associated with saturation of mental health services with either greater levels of risk or higher levels of reporting activity.^10^

Our findings showed how the complexity of the wider system can influence the processes and outputs from the patient safety processes. In addition to highlighting the inherent complexity of the system, participants identified both advantages and disadvantages of its multiple interdependent elements. The advantages included the incorporation of different perspectives, ensuring information and/or learning was not missed, and providing a broader view of the incident(s). The disadvantages included potential cascading failures during the investigation processes due to the complexity of the interdependency inputs.

### Implications for clinicians and policymakers

A critical aspect of the patient safety system that has been empirically investigated is patient safety culture.^23^,^25^ Healthcare policymakers in the UK have considered culture change to be as important as structural modification of the patient safety system, with organisations striving for an open culture—where staff feel able to report incidents/concerns—and a ‘just’ culture, in which incidents are investigated and responded to fairly, without blame and with the facilitation of learning.^26^ Effecting cultural change within an organisation requires the activation of processes that lead to a system-wide shift in assumptions, attitudes and beliefs.^26^ As shown in the findings of our study, there is an expectation that the newly adopted system-based approach to investigation will positively influence safety culture through a change in staffs’ assumptions, attitudes and beliefs. However, our findings suggest that, despite the move towards systems-based investigations, individuals may still experience feelings of blame, accountability may shift to different parts of the system and certain elements within the system continue to emphasise individual accountability.

Our findings are similar to other research conducted in general healthcare, where senior organisational patient safety meetings focused more on practical information, such as the direct cause of an incident, rather than systems aspects.^27^ This failure to focus on and implement system-based findings and learning may cascade down the pathway to the professional workforce, resulting in feelings of blame. Other qualitative studies have recognised that a clear hierarchical structure combined with a sense of duty and care towards frontline clinicians downstream of management can ensure psychological safety when learning from mistakes.^27^ Organisations should make sure system-based factors remain at the forefront and that senior leaders are equipped to cascade these systems-based findings in a psychologically safe method to frontline clinicians. If there are significant feelings of blame, it is likely to impact on efforts to improve the safety and quality of care delivered by those professionals at the micro-level (ie, frontline).^22^ If there are differing expectations from elements of the system that are contradictory to an organisational ‘no-blame’ culture or a drive for systems-based findings, this has the potential to seriously damage trust between workers and the organisation.^8^

Other studies have found that the ‘no-blame’ systems approach has been criticised for potentially absolving responsibility from individual human error.^28^ This was one of our findings, identified by participants as a potential negative impact of system-based investigation. The alternative argument is that by understanding the healthcare system, fundamental questions about the people involved, the wider system and its risks can be addressed and mitigated.^29^ Regardless of the approach to investigating an incident, involvement in a patient safety event or incident all places the individual, be that professional or patient, within the complex healthcare system being investigated.^30^ This means that although the primary intention of the incident investigation is to view an incident as a system fault in which an individual is present, an element (eg, trust executives) within the complex system can change expectations and functions within the incident response pathway. This was a thematic finding in our study where elements in the system can introduce a concentration on individual errors or linear causality, despite a shift to systems-based investigations. This alteration could lead to a drive for more individual accountability, feelings of blame and reduced willingness to raise concerns. This could all result in a negative impact on safety culture, despite the introduction of a system-based approach to investigation.

Our results are similar to other studies, where the pursuit of a no-blame culture in safety in mental healthcare is likely to be a chimaera, where organisations may need to evolve and adapt to consider how the workforce on the ground is likely to retrospectively feel blamed, and to anticipate negative consequences of blame.^13^ Clinical practice and future research should seek feedback from the frontline workforce, in addition to incident investigators and safety managers, during changes in patient safety strategies. This may help to identify any unintended and/or undesirable consequences of the changes made within a complex healthcare system.^11^ A better understanding of how risk and safety are operationalised within real-world complex healthcare systems will help inform improvements in patient safety while enabling the identification of unintended consequences for healthcare professionals, with a view to mitigating them.

### Considerations for future research

Research has a role in developing a greater understanding of the highly interconnected technical and social entities that dynamically produce emergent behaviour within the patient safety system. Our study shows that social entities (eg, external bodies, trust executives) can shift technical safety strategies and their outcomes. There is little (if any) research that describes or maps the patient safety system within mental healthcare, considering its elements and interfaces. Our study provides an important foundation on which to build. This research could be expanded on further to capture the systemic effects, combining an organisational ethnographic methodology with a systems mapping approach. Systems thinking and complexity science could be used to develop a systems model/map, which could be enriched further with quantitative inputs. This system map can then provide a model of when and how different elements interact and influence the function of safety incident investigation. This could develop an understanding of how safety policy is implemented and operationalised across the multi-level system. Additionally, without a nuanced understanding of the whole complex patient safety system, potential adverse consequences of well-meaning initiatives localised in one part of the system may not be identified until their effect in compromising patient safety has occurred.

### Strengths and limitations

The timing of the interviews was both a benefit and a limitation, where the two NHS trusts were both in the early stages of implementing PSIRF. The benefits are that this allowed for a more accurate comparison between the new safety strategy and previous approaches to incident investigation. It also provides vital data for healthcare organisations and for patient safety teams who are intending to, or have recently, implemented a systems-based approach to incident investigation. A limitation is that the participants interviewed may have limited experience in using the new framework where there is some uncertainty about how best to operationalise the process and how effective it is.

This study used different forms of sampling, embedded expertise into the sampling and data collection processes, and considered the sample size iteratively as the study progressed. However, what was evident as the research progressed is the complexity of the system, with a myriad of interdependencies and elements that interact. This means that more stakeholders could have been interviewed due to the vast nature of the system and its interactions. This may have included leads in education and training, board executives and those from independent bodies such as the Healthcare Safety Investigation Branch. Snowball sampling within interviews could be included to identify elements that less frequently interact. Additionally, although we interviewed frontline clinicians, this included those with additional leadership responsibilities. This may have missed perceptions of what this systemic change in adverse incident responses means for most clinicians. Future research, such as that described above, should aim to address this limitation and create a detailed map of this system.

## Conclusions

The NHS patient safety strategy puts system-based investigation and learning at its heart. Our study demonstrates that those working within the safety system in mental healthcare value this shift, with the hope that systems-based investigations will reduce feelings of individual blame and promote an improved safety culture. However, findings suggest that our limited understanding of the safety system in mental healthcare introduces complexity and uncertainty to the functions of incident investigation. The processes and outcomes of systems-based investigations can be influenced by different elements of the system in incident investigation, which could impact frontline clinicians’ feelings of blame and on organisational safety culture. Acknowledging and evaluating this complexity through appropriate methodologies is likely to reduce any potential negative outcomes that exist. Realising the essence of the patient safety strategy necessitates a sustained dedication to cultural transformation (for systems-based reporting, investigating and improvement), robust authentic accessible leadership and embedded sustainable feedback loops for effective learning.

## Supplementary material

10.1136/bmjopen-2025-104863 online supplemental file 1

10.1136/bmjopen-2025-104863 online supplemental file 2

10.1136/bmjopen-2025-104863 online supplemental file 3

10.1136/bmjopen-2025-104863 online supplemental file 4

## Data Availability

No data are available.
