# Supplementary material for: Understanding adverse incident responses in mental health care: a qualitative study of systems-based patient safety practices
Source: BMJ Open. 2025 Nov 9;15(11):e104863. doi: 10.1136/bmjopen-2025-104863 (PMC12598960; doi:10.1136/bmjopen-2025-104863)
Supplement: online supplemental file 2 [file bmjopen-15-11-s002.docx]

**Supplementary Material B**

Interview Schedule

This is an exploration of your role and understanding of the patient safety system and the approaches to incident investigation. We are interested in your experiences of the processes within the patient safety system in mental health care, and your experiences with different methods of investigating incidents.

For each system element, a description will be gathered of the role of the individuals and the team as it relates to the incident, the action taken, and the lines of communication. These discussions will be guided by a semi-structured proforma which will focus on the processes, roles, ways of seeking assurance, and determining accountability and responsibility. The interviewer will also explore for unintended consequences of the process.

The following prompts will be used to encourage relevant discussions but should not be delivered rigidly.

1. Role

- What is your role?
- How does this role sit within the patient safety system?
- Do you focus on one element of patient safety within your role?

1. Patient Safety

- What does patient safety mean to you?
- How is patient safety monitored within your organisation?
- What are the potential areas of patient harm?
- What do you see as the patient safety culture within the NHS?
- How do you see safety culture in your workplace?

1. Incident Investigation

- Can you talk me through your understanding of incident investigation approaches, explore RCA, systems, transition to PSIRF.
- Do you see patient safety responses are viewed as a system or an orientation that finds and attaches blame to individuals?
- Are we using recognised system-based methodologies for data collection and analysis?
- How do we choose our response to a patient safety incident?
- How do we know how well our processes are working?
- Have you been involved in a patient safety incident? How did you feel? How did the organisations response make you feel? Did any of the processes or involvement of the organisation impact the outcome?
- Can you talk me through the different teams that are involved in incident investigation? How do they influence the investigation and its outcome?
- What are the roles, responsibilities, and processes for oversight within providers and with the ICB? What influence do they have on incident investigation? Do other external factors influence?

1. Learning

- What follows on from the incident investigation?
- How does the place you work try to improve patients’ safety? What systems / processes are in place?
- How are the learning responses resourced (including funding, time, equipment, and training)?

1. Patient/family/carer involvement

- How do you Engaging and involving patients, families in patient safety?
- Are patients or staff with protected characteristics represented more often than others in any of our incidents and responses? What are the organisational or cultural reasons behind this?
