# Supplementary material for: Understanding adverse incident responses in mental health care: a qualitative study of systems-based patient safety practices
Source: BMJ Open. 2025 Nov 9;15(11):e104863. doi: 10.1136/bmjopen-2025-104863 (PMC12598960; doi:10.1136/bmjopen-2025-104863)
Supplement: online supplemental file 3 [file bmjopen-15-11-s003.docx]

**Supplementary Material C – Positioning and Reflexivity**

The research team includes the lead author, a clinician-academic and consultant in Forensic Psychiatry, who has experience in qualitative research. He was employed within one of the clinical services studied. The lead author conducted interviews and independently analysed data. An experienced clinician-academic and senior leader, RN, employed in one of the participating organisations, also conducted interviews and independently analysed data. A doctor in training was present during those interviews. The remainder of the research team includes a mental health nurse and academic, experienced mixed-methods researchers in patient safety, a director of patient safety, and a researcher of complex systems and systems thinking.

Positioning and reflexivity were considered within the study. Where possible, the interviewers attempted to recruit and interview participants where they had no prior relationship with the study participants, to reduce the influence of the researcher on the interview. Due to the senior leadership status of one of the data collectors within a service studied, this was not always possible. Where not possible, this prior relationship was considered within data collection and analytic procedures. Within those interviews an additional researcher was present, to observe relational dynamics and influences, which could then be used reflexively to guide future interviews and for data analysis.

As frontline clinicians, those collecting, analysing and writing up data considered their past experiences within incident investigations and how that may have influenced the interviews and study findings. The authors considered the cultural values within the social environment of the clinical service, reflected on the relational influence within interviews of the aspects of the social identity (e.g., clinical role, academic expertise) and how their experiences of working as frontline clinicians may influence interviews with those in managerial role in the services.

A reflexive diary was completed to allow the researchers to reflect on positioning and influence within the study research process. The research team met regularly to review interview progress, and to consider alternative perspectives and interpretations. To further minimise and moderate researcher influence on data collection and analysis, a description as close to the participants views as possible was gathered.
