# Supplementary material for: Understanding adverse incident responses in mental health care: a qualitative study of systems-based patient safety practices
Source: BMJ Open. 2025 Nov 9;15(11):e104863. doi: 10.1136/bmjopen-2025-104863 (PMC12598960; doi:10.1136/bmjopen-2025-104863)
Supplement: online supplemental file 4 [file bmjopen-15-11-s004.docx]

**Research Proposal**

**Title:** Researching patient safety in a healthcare system: a project to enhance understanding of patient safety processes across a complex system and to develop a mixed methods approach to empirically characterise the complex system.

**1.0 Introduction**

Healthcare providers and systems expend a great deal of effort and resource on improving patient safety, the goal of which is to reduce the occurrence of avoidable harm. Whilst the radical change in approach to patient safety recently recommended by NHS(E) is underpinned by an intention to deal with issues that have previously been identified as having frustrated efforts to reduce harm, the evidence base supporting the change is very limited. To develop the evidence base, patient safety as understood within health systems should be seen an intervention that involves varied processes taking effect across a complex system that in combination should reduce avoidable harm. The complexity can be illustrated by the emphasis in the new strategy on cultural issues within the organisation (e.g., investigating patient safety incidents within the context of a ‘just culture’).

To effect cultural change within an organisation requires the activation of processes that lead to a system-wide shift in assumptions, attitudes, and beliefs. Moreover, the new strategy calls for an approach to investigating patient safety incidents that is system-based, another concept that is manifest diffusely across the system. Those receiving and delivering care in mental health services face similar risks to those in other areas of health care. However, the behaviours associated with serious mental illness (e.g., self-harm and/or violence to others) and the interventions aimed to manage these (e.g., risk assessment tools, restraint) add further layers of complexity to patient safety. The goal of a patient safety strategy is to allow healthcare needs to be addressed whilst reducing avoidable harm, but whether this goal is being achieved has not been robustly evaluated.

Secure and forensic mental health services provide specialist care for patients detained under the Mental Health Act because their mental disorder is perceived as posing a risk to themselves or to others. The concept of risk and patient safety is the raison d’etre for the structure and operationalisation of secure services. Patient safety can often be approached as the inverse of risk in these services, which may neglect the aim of patient safety, a discipline that aims to reduce avoidable harm in healthcare, developed in parallel with the increasing complexity of healthcare systems.

This project aims to focus on the complex system of secure mental health services. Understanding the combined effect of interacting patient safety processes across the complex system of secure mental health services is important due to its inherent complexity and its totalising focus on risk. Within healthcare services the notion of risk is predominantly linked to patient outcomes. In secure mental health care, there is a lack of empirical understanding of patient outcomes with a potential for decision-making that necessitates conflicts between legal, ethical, clinical, and societal values and may lead to disproportionate risk aversion.

The project leads have produced empirical evidence to show that, in day-to-day practice, healthcare professionals are not only considering patients’ clinical and risk needs, but that they routinely contemplate how their decision-making will be scrutinised in the event (however unlikely) of a patient safety incident. This may lead them to amend their practice to address the needs of the organisation’s patient safety strategy (even if the amended practice may negatively impact on patient outcomes and their own wellbeing). A better understanding of how risk and safety is operationalised within real world complex healthcare systems will not only inform improvements in patient safety, but it will also mean that unintended consequences for healthcare professionals can be identified with a view to mitigating them. A greater understanding will allow for the development of digital technologies and learning and training opportunities in patient safety.

The National Health Service has begun to recognise the need for this, with the Patient Safety Strategy attempting to promote a patient safety culture and patient safety system. It highlights the need for ‘insight’, an approach to improve understanding of safety by drawing from multiple sources of patient safety information. The new Patient Safety Incident Response Framework (PSIRF) aims to support insight generation at the point of care, applying a range of system-based approaches to learning from patient safety. The Health Car Safety Investigation Branch are also dedicated to improving patient safety across the NHS(E) with a focus on systems and processes in healthcare.

Risk management culture has previously focussed on self-harm, suicide, and violence, whilst the new NHS strategy PSIRF focusses on the processes within the healthcare system itself. The evidence base suggests that the poorly defined nature of patient safety in mental health care can create challenges for patient care, with NHS psychiatrists lacking aware of the wider safety context and not recognising certain types of potential safety incidents. Thus, simply transitioning to a new model of incident reporting and investigation is unlikely to lead to meaningful change without a greater understanding of the nature of patient safety in mental health services.

Work undertaken by the project team has shown that this shift alone may not address the factors that interfere with the operationalisability of learning for everyday practice. For example, an investigation into a patient safety incident will interface on one hand with the service where the patient incident occurred (i.e., in direct contact with patients) and on the other hand through other people and forces, which may be removed in time and space from direct care, but nonetheless affect how the care is delivered. These many layers of the complex system include wider managerial and governance structures that form part of the organisation. Through this structure, a sequence of processes is activated, some determined by relevant operating procedures and policy and through less formalised parts of the system. These approaches inform a system-based process to investigation, but little is known about how risk and patient safety is conceptualised within health care levels (e.g., organisational, social, individual), their sub-components (e.g., policies, management decisions, technological factors), and how they interact and link. Research has not investigated the dynamics that exist across the complex system and the interconnections between decisions, policies and change at one level of analysis with others.

A framework that has been used within the field of implementation science involves three levels of intervention, that have been theoretically explored through frame analysis. These levels include (i) macro level, system wide approaches with a focus on strategy/policy/infrastructure, (ii) meso level, the organisation levels involving local systems, pathways, services, (iii) micro level comprising the patient level.

**2.0 Aims**

The objectives of this project are (i) to undertake a focussed literature review relevant to evaluating patient safety systems in mental health care, (ii) to engage relevant stakeholders to produce a preliminary map of the elements and interfaces of the safety system in a forensic mental health setting, and (iii) to work with methodologists to reach a robust mixed methods methodology to evaluate a complex safety system. This second objective will be achieved by interviews with operational stakeholders across an integrated care board (ICB) in the northwest of England (NHS Cheshire and Merseyside). The resulting preliminary map will then form the focus of the collaboration with qualitative and quantitative methodologists in the field of complex systems (who are part of the project team). It is proposed that the applicant will work further with those experts to develop a robust approach to the evaluation of complex safety systems across all of mental health care, to formalise a detailed system map, describe how the system is operationalised, and consider how the current approach could work better.

**3.0 Methods**

3.1 Study Design

The initial phase of the research is through a narrative review of the literature. The second phase is qualitative semi-structured interviews with stakeholders. The third phase is the mapping of the patient safety system and development of research methodology to investigate the mapped patient safety system.

*3.1.1 Literature Review*

A narrative review was chosen to enable a broader exploration of topics. The search will focus on mental health care and the wider fields of patient safety and implementation science. Articles will be purposively selected and their conceptual contribution will be discussed with the project team and steering group.

Key aspects of the search include:

- Patient safety – defined by the World Health Organisation definition, the prevention of errors and adverse effects to patients associated with health care.

- Mental health – Mental health is defined as a healthcare service comprising various professions that manages the mental and psychological well-being and the prevention, diagnosis or treatment of mental disorders as listed in the Diagnostic and Statistical Manual fifth edition.

- Forensic / Secure mental health – A specific sub-focus will be on secure mental health services. Secure mental health care is focussed on the treatment of mentally disordered offenders.

- Healthcare Systems -

There is no definition of setting. A large proportion of mental health and patient safety research has focussed on the inpatient setting. This research will have a broader review of all secure mental health services.

A proportion of research will include interventions for patient safety, for example interventions to reduce suicide risk, violence risk. Papers will be included if they include patient safety outcomes. Research conducted on patient safety interventions that focus only on validity/reliability of the tool/intervention will be excluded.

Further exclusion criteria for the search will consist of:

- A population that is defined as outside the remit of mental health services, e.g., physical healthcare

- Papers not in English.

*3.1.2 Qualitative Semi-Structured Interviews*

Semi-structured interviews will be conducted with professionals within mental health services. The stakeholders will be identified through the literature review, via liaison with a steering group and the project team, and via the qualitative interviews undertaken.

A semi-structured interview will be designed by authors (A.C and R.N). The interview format will cover areas of; (i) roles within the patient safety system, (ii) operationalisation and processes within the system, (iii) identification of others roles within the system and how they interact.

The interview guide will remain flexible, informed by emerging results from the wider body of work. Interviews are not expected to last longer than one hour.

*3.1.3 Patient Safety Mapping and Research Methodology Development*

The qualitative data will be used to create a preliminary map of the patient safety system. The nature of the patient safety system within mental health care will be defined and liaison with complex system methodologists will then develop a research approach and methodology for a larger project investigating the patient safety system in mental health care. Developing a patient safety process map will draw upon principles of system mapping that have been used in patient safety research in physical health care. The mapping of patient safety systems in mental health care has not been conducted.

3.2 Recruitment

Stakeholders working within mental health services will be invited to take part in the study.

The study team will aim to interview participants from a range of secure services and within different NHS Trusts across the North West of England. A varied sample of stakeholders will be used across different setting to have a wider view of the patient safety system.

A steering group of PPI representatives within the project teams organisations will be used to to provide an expert oversight and ensure a patient/carer perspective guides the recruitment. It will also discuss how patients/carers are involved within the system and explore their perspective on the stakeholders within the patient safety system. Patient/carers will not be interviewed at this stage of the research.

Stakeholders will also be recruited using purposive and snowballing sampling, via the approach of using the researchers existing clinical networks of professionals. The semi-structured interview will reveal further potential recruits, and these will be approached accordingly to ensure a comprehensive map of the system is developed.

The specific stakeholders that will be approached for interview includes directors of patient safety, clinical and operational managers, frontline clinicians/nurses/psychologists, Integrated Care Boards PSIRF leads, commissioners, policy leads, independent patient safety organisation representatives, coroners, care quality commission representatives, external stakeholders through patient groups and patient and public representative organisations, and internal stakeholders whose divisions/services with patient safety processes e.g., educational, governance etc. an integral aspect of this project is the identification of stakeholders within patient safety system. Having a wider open remit will allow identification of stakeholders that one might not expect to be involved. This is key to successfully map and examine the patient safety system.

Potential participants will be approached using an introduction letter and an information sheet. Additionally, they will be invited to contact the research team to discuss implications for participating or for further information.

Professionals will be invited to take part in the interviews at a time and location that is convenient for them. Interviews will be carried out in person and/or using virtual video software, which will improvement recruitment.

3.3 Data Collection

Data will be collected via the use of audio recording devices and field notes. Participant consent will be sought to audio record the interviews. Data will be transcribed and anonymised. No personal data will be collected as part of the interviews or observations. All direct quotes and notes will be fully anonymised and contain no person identifiable information. The data collected will then be analysed by the research team.

3.4 Data Analysis

Data will be analysed using a framework method. The Framework Method for the management and analysis of the qualitative data involves seven stages (Gale et al., 2013):

Stage 1: Transcription

Stage 2: Familiarisation with the interview

Stage 3: Coding

Stage 4: Developing a working analytical framework

Stage 5: Applying the analytical framework

Stage 6: Charting data into the framework matrix

Stage 7: Interpreting the data

This approach will produce a matrix of summarised data (stage 6), which can then provide a structure to analyse, interpret and reduce the data. A benefit of this qualitative method is that the context of participants data is not lost. To add methodological rigour, a second researcher will independently analyse a proportion of the data to check accuracy of coding and the framework. Additionally, this framework and the extracted data will be discussed by the research team to ensure a collaborative process.

The qualitative data will then be used to create a preliminary map of the patient safety system. The nature of the patient safety system within mental health care will be defined and liaison with complex system methodologists will then develop a research approach and methodology for a larger project investigating the patient safety system in mental health care.

3.5 End of Study

The end of study will be the completion of the data analysis of the qualitative data, and the subsequent use of that data. This data will be used to map the patient safety system and to consider quantitative methodological approaches in future studies. We anticipate this to be 1-2 months following completion of data collection from the interviews and observations.

3.6 Ethics

The main ethical considerations for this study relate to informed consent and confidentiality.

Direct quotes and notes used within interviews and observations will be fully anonymised and contain no identifiable information. Participants will be provided with an information sheet detailing the use of anonymised, direct quotations, what data is being collected, how it is being collected, data storage, and the limits of confidentiality. Informed consent will be sought on the participants involvement in the research and specifically for the use of anonymised, direct quotations.

Participants will be asked consent to the use of audio device recording equipment. As noted above, all transcripts will be fully anonymised. Once the audio recorded data is transcribed, the audio files will be permanently deleted.

Data will be stored as electronic files on password protected NHS Trust computers, on secure NHS servers. Paper field notes will be securely stored in lockable filing cabinets on NHS Trust premises. Any consent forms and participant information sheets will be stored separately to the study data. Data will be stored for three years.

Discussions regarding patient safety can involve sensitive topics related to patient violence and self-harm. Participants will be advised that they can stop the interview or observations at any time. They will also be reassured that they do not have to answer all questions posed to them. I note that it is likely that all participants have experience of working within patient safety and mental health services. Any support in case of distress will be provided.

I do not consider there to be any specific risk to the research team. The researchers have experience in conducting research in the field of patient safety. The team will meet regularly, and any concerns will be reflected on and discussed.
