# Supplementary material for: Understanding adverse incident responses in mental health care: a qualitative study of systems-based patient safety practices
Source: BMJ Open. 2025 Nov 9;15(11):e104863. doi: 10.1136/bmjopen-2025-104863 (PMC12598960; doi:10.1136/bmjopen-2025-104863)
Supplement: Supplementary file 5 [file bmjopen-2025-104863.reviewer_comments.pdf]

PEER REVIEW HISTORY

BMJ Open publishes all reviews undertaken for accepted manuscripts. Reviewers are asked to complete a checklist review form and are provided with free text boxes to elaborate on their assessment. These free text comments are reproduced below.

ARTICLE DETAILS

Title (Provisional)

Understanding Adverse Incident Responses in Mental Health Care: A Qualitative Study of Systems-based Patient Safety Practices

Authors

Challinor, Alexander; Berzins, Kathryn; Bifarin, Oladayo; Anderson, Nina; Xavier, Panchu; Saini, Pooja; Morasae, Esmaeil Khedmati; Nathan, Rajan

---

VERSION 1 - REVIEW

**Reviewer**	**1**
**Name**	**Chakraborty, Nandini**
**Affiliation**	**Leicestershire Partnership NHS Trust**
**Date**	**15-Jul-2025**
**COI**	**None**

---

1. The study objectives do not define specific questions and overlaps with points which should be mentioned in design and methodology. Engaging stakeholders in a semi-structured interview is part of the method not the objectives. The objectives should include clear questions which the research tries to address and find answers to.

2. The title mentions that this is a qualitative study. The qualitative aspect has not been mentioned in the abstract. I think this is an important point to be mentioned in the design in the abstract.

3. The results and conclusions in the abstract are presented in a convoluted way which makes it difficult to extract clinically relevant information. What are the advantages and disadvantages identified? The impact of the transition to a system-based approach is mentioned in the aims but does not feature in the results or conclusions. The abstract could be better presented by mentioning the main findings within the themes, which are more distinct and detailed in the main write up.

'Participants in this study reported that the change in approach to incident investigation is likely to lead to beneficial change. The systems approach to investigation was thought to be more robust and more effectively linked to learning. There were considerations of potential

negative effects from a systems-based approach, such as the shifting rather than elimination of blame, and the possibility of missing individual poor practice'. This is a key paragraph which is worth summarizing in the abstract.

4. The themes which emerge from the interviews with quotes is the most well-written part of the paper. The directions of future research are enveloped in technical jargon which make difficult reading for the frontline clinician affected by investigations.

5. The final conclusion in the paper does not focus on the transition from root-cause analysis to a systems-based approach. This is supposed to be the main essence of the paper. The conclusions remain vague. A clear conclusive statement about how the interviewees felt about the transition would be helpful.

6. Table 1 lists 20 professionals instead of 19 as mentioned everywhere else.

7. I also think that it is a limitation of the study that it does not include frontline clinicians, or if it does this is not clear. Are their clinicians without a managerial job included- perceptions of what this systemic change in adverse incident responses means for the vast majority of clinicians?

Overall, the study addresses an important topic and strives to explore systemic changes in adverse incident responses, an important aspect influencing clinical practice and how clinicians perceive their working environment. 19 interviews providing qualitative data is a robust amount of information. However, the conclusions and findings could be presented a lot more clearly and relevantly for clinicians.

**Reviewer**	**2**
**Name**	**Woodnutt, Samuel**
**Affiliation**	**University of Southampton**
**Date**	**30-Jul-2025**
**COI**	**None**

Many thanks for the opportunity to review this manuscript. On the whole, the work addresses a key and contemporaneous issue in modern mental health care. I have a few comments to make, and have made a number of suggestions on the attached manuscript. Overall, I do believe that there is a robust and decent study underneath this, however the write up seems to be severely letting it down and making it very difficult to actually interpret any of the results in light of the lack of clarity in methods. This leads to the view that the study is prone to bias - although, I believe it was probably appropriately executed - it is not clear.

It is not clear, and there is no consideration in the write up, of whether ethical approval was sought and granted for the study - either institutional or NHS related. This needs to be rectified for a study purporting to be qualitative in nature.

One major comment, is that the introductory paragraphs are particularly under-referenced. I am unsure if this is the author's intention to limiit the reference list to 20, but I think this could be increased for the amount of ground being covered, and the complexity of the discussion. Whilst some of the seminal texts have been noted (e.g. Thibault et al.), there are other assertions made without references which would be easily strengthened with a citation. The overall effect of this is that much of the introduction is somewhat speculative in nature, and not clearly grounded in research. This needs to be addressed to therefore strengthen the overall work.

In methods, whilst ostensibly robust, there are some wording issues that need rectifying to be able to provide a clear audit trail of how the participants were identified and then recruited - I have made a number of comments to the methods to seek clarification for this, as at present it is not clear and therefore leaves interpretation of the methods at high risk of bias without being able to consider the detail.

Essentially, the methods section needs to be more or less like a recipe where others would be able to replicate - at the moment it is a partial log of what happened during the development of the study, rather than a robust set criteria. My strong suggestion is to consider the using the COREQ tool over the SRQR tool as COREQ is designed for qual research in a health setting, whereas the SRQR is quite broad, and seems to have been interpreted with minimal amounts of depth. COREQ may facilitate the authors to report the level of detail required when reporting robust studies.

For these reasons, I have not given the Results and Discussion sections a great amount of focus, as they are difficult to interpret when there are the unknowns from the methods. From my view of this, they generally seem well written, and include a range of illustrative quotations from participants. The main limitation with Results at this stage is that the thematic analysis seems somewhat superficial, and lacks the amount of depth in exploration of themes normally expected of studies of this size. I am also not convinced that heading the results with positives and negatives is the most compelling way to present the results - whereas the latter themes are more sophisticated, and therefore more compelling...

**** The reviewer provided a marked copy with additional comments. Please contact the publisher for full details..pdf ****

VERSION 1 - AUTHOR RESPONSE

Reviewer: 1

Dr. Nandini Chakraborty, Leicestershire Partnership NHS Trust

Comments to the Author:

1. The study objectives do not define specific questions and overlaps with points which should be mentioned in design and methodology. Engaging stakeholders in a semi-structured interview is part of the method not the objectives. The objectives should include clear questions which the research tries to address and find answers to.

The research aims/objectives have been more succinctly defined to add clarity and avoid overlap with methodology.

2. The title mentions that this is a qualitative study. The qualitative aspect has not been mentioned in the abstract. I think this is an important point to be mentioned in the design in the abstract.

The qualitative design of the study has been added into the abstract.

3. The results and conclusions in the abstract are presented in a convoluted way which makes it difficult to extract clinically relevant information. What are the advantages and disadvantages identified? The impact of the transition to a system-based approach is mentioned in the aims but does not feature in the results or conclusions. The abstract could be better presented by mentioning the main findings within the themes, which are more distinct and detailed in the main write up.

'Participants in this study reported that the change in approach to incident investigation is likely to lead to beneficial change. The systems approach to investigation was thought to be more robust and more effectively linked to learning. There were considerations of potential negative effects from a systems-based approach, such as the shifting rather than elimination of blame, and the possibility of missing individual poor practice'. This is a key paragraph which is worth summarizing in the abstract.

Thank you for highlighting a key paragraph that could be used in the abstract to better portray the main findings from the study. The abstract has been amended to present a clearer and more informative picture of the study findings.

4. The themes which emerge from the interviews with quotes is the most well-written part of the paper. The directions of future research are enveloped in technical jargon which make difficult reading for the frontline clinician affected by investigations.

The directions for future research has been amended to improve readability. Thank you for the feedback on the writing of the results section.

5. The final conclusion in the paper does not focus on the transition from root-cause analysis to a systems-based approach. This is supposed to be the main essence of the paper. The conclusions remain vague. A clear conclusive statement about how the interviewees felt about the transition would be helpful.

The feedback on providing a clear conclusive statement has been actioned, including this within the conclusion section. The conclusion has been made more focussed.

6. Table 1 lists 20 professionals instead of 19 as mentioned everywhere else.

Apologies for this error, this has been amended accordingly.

7. I also think that it is a limitation of the study that it does not include frontline clinicians, or if it does this is not clear. Are their clinicians without a managerial job included- perceptions of what this systemic change in adverse incident responses means for the vast majority of clinicians?

Yes, frontline clinicians with additional managerial responsibilities were recruited. This has been made clearer in the results section. I have also included an additional sentence in the strengths and limitations section that details this as an important limitation, thank you for highlighting this.

Overall, the study addresses an important topic and strives to explore systemic changes in adverse incident responses, an important aspect influencing clinical practice and how clinicians perceive their working environment. 19 interviews providing qualitative data is a robust amount of information. However, the conclusions and findings could be presented a lot more clearly and relevantly for clinicians.

Thank you for the helpful feedback on the manuscript, which has refined and improved the conclusion and findings. We hope that with the helpful suggestive changes the findings and conclusions are more relevant for clinicians.

Reviewer: 2 (see also doc attached)
Dr. Samuel Woodnutt, University of Southampton

Comments to the Author:

Many thanks for the opportunity to review this manuscript. On the whole, the work addresses a key and contemporaneous issue in modern mental health care. I have a few comments to make, and have made a number of suggestions on the attached manuscript. Overall, I do believe that there is a robust and decent study underneath this, however the write up seems to be severely letting it down and making it very difficult to actually interpret any of the results in light of the lack of clarity in methods. This leads to the view that the study is prone to bias - although, I believe it was probably appropriately executed - it is not clear.

Thank you for the informative feedback on the study. The write up has been amended to add greater detail to the methodology. The refinement of the results/discussion has freed up sufficient word count to allow greater detail to be added into the methods, and to provide richer descriptions as per COREQ and SRQR. This has improved the reliability, transparency and replicability of the study. More specifically, a section on study design has been added to further describe the qualitative approach and research paradigm. A supplementary material has been added to describe positioning and reflexivity further, hopefully addressing aspects of bias and how these were controlled for. The recruitment steps are detailed greater, with considerations for sampling rationale, factors that may have influenced findings, and strategies to reduce bias.

It is not clear, and there is no consideration in the write up, of whether ethical approval was sought and granted for the study - either institutional or NHS related. This needs to be rectified for a study purporting to be qualitative in nature.

Ethics statement/ref has been included. It was on the title page in the initial submission owing to the blinded peer review process, I have added it into the methods.

One major comment, is that the introductory paragraphs are particularly under-referenced. I am unsure if this is the author's intention to limiit the reference list to 20, but I think this could be increased for the amount of ground being covered, and the complexity of the discussion. Whilst some of the seminal texts have been noted (e.g. Thibault et al.), there are other assertions made without references which would be easily strengthened with a citation. The overall effect of this is that much of the introduction is somewhat speculative in nature, and not clearly grounded in research. This needs to be addressed to therefore strengthen the overall work.

Relevant cited material has been included, with the more speculative statements re-worded and grounded in research. The highlighted speculated paragraph has been removed from the introduction, condensed and placed into the discussion. Thank you for highlighting sentences that would benefit from citations, these have been added.

In methods, whilst ostensibly robust, there are some wording issues that need rectifying to be able to provide a clear audit trail of how the participants were identified and then recruited - I have made a number of comments to the methods to seek clarification for this, as at present it is not clear and therefore leaves interpretation of the methods at high risk of bias without being able to consider the detail.

The comments on the manuscript have been followed and amended accordingly, thank you. The method includes a stepwise guide to recruitment and the further steps of the method process.

Essentially, the methods section needs to be more or less like a recipe where others would be able to replicate - at the moment it is a partial log of what happened during the development of the study, rather than a robust set criteria. My strong suggestion is to consider the using the COREQ tool over the SRQR tool as COREQ is designed for qual research in a health setting, whereas the SRQR is quite broad, and seems to have been interpreted with minimal amounts of depth. COREQ may facilitate the authors to report the level of detail required when reporting robust studies.

A greater level of detail has been added throughout the methods, thank you. This hopefully reads more like a replicable recipe. The SRQR has been followed as per journal requirements. This guideline also aligns better with the reflexive, interpretive approach, where COREQ can align more closely with realist of post-positivist approaches.

For these reasons, I have not given the Results and Discussion sections a great amount of focus, as they are difficult to interpret when there are the unknowns from the methods. From my view of this, they generally seem well written, and include a range of illustrative quotations from participants. The main limitation with Results at this stage is that the thematic analysis seems somewhat superficial, and lacks the amount of depth in exploration of themes normally expected of studies of this size. I am also not convinced that heading

the results with positives and negatives is the most compelling way to present the results - whereas the latter themes are more sophisticated, and therefore more compelling...

Thank you for this feedback, as noted by reviewer 1 and 2, the findings were well written, interesting and appropriately quoted. Upon re-reading we would agree with the lack of depth within theme 1, which was potentially trying to describe a richly explored area in one theme, leading to the perception of the result being banal or superficial. To resolve this issue, we have expanded the subthemes found into two themes, one exploring the participants perceptions of the differences between incident investigation approaches, and one describing the perceived redistribution of accountability as a result of the change in approach. Given the analytical findings and positive feedback from both reviewers for the latter more compelling themes, these have been maintained. I have included a summary box at the beginning of the results to also succinctly describe each theme to aid readability.

We have added a paragraph within the main findings of the discussion to better place the findings within the wider literature and within methodological factors relevant to the study.

Thank you again for the feedback, we are of the opinion it has improved the manuscript.

Reviewer: 1
If you have selected ‘Yes’ above, please provide details of any competing interests.: Not applicable

Reviewer: 2
If you have selected ‘Yes’ above, please provide details of any competing interests.: Not applicable

---

VERSION 2 - REVIEW

**Reviewer**	**1**
**Name**	**Chakraborty, Nandini**
**Affiliation**	**Leicestershire Partnership NHS Trust**
**Date**	**24-Sep-2025**
**COI**	

The authors have addressed all reviewer comments adequately. I have no further suggestions to make.

Reviewer 2
Name Woodnutt, Samuel
Affiliation University of Southampton
Date 07-Oct-2025
COI

Thank you for this much revised and polished manuscript. The comments raised in previous reviews have been addressed, and there are a number of improvements which clarify the methods and therefore the reporting as a whole. I belive this is a valuabel and timely study which will add to the evolving picture of evidence.
